# Genome-Wide Diversity Analysis of African Swine Fever Virus Based on a Curated Dataset

**DOI:** 10.3390/ani12182446

**Published:** 2022-09-16

**Authors:** Jingyue Bao, Yong Zhang, Chuan Shi, Qinghua Wang, Shujuan Wang, Xiaodong Wu, Shengbo Cao, Fengping Xu, Zhiliang Wang

**Affiliations:** 1 State Key Laboratory of Agricultural Microbiology, College of Veterinary Medicine, Huazhong Agricultural University, Wuhan 430070, China; 2 China Animal Health and Epidemiology Center, Qingdao 266032, China; 3 Lars Bolund Institute of Regenerative Medicine, Qingdao-Europe Advanced Institute for Life Sciences, BGI-Qingdao, BGI-Shenzhen, Qingdao 266555, China; 4 College of Life Sciences, University of Chinese Academy of Sciences, Beijing 518083, China; 5 BGI-Qingdao, BGI-Shenzhen, Qingdao 266555, China

**Keywords:** African swine fever virus, genome sequence diversity, curated dataset, tandem repeat sequences

## Abstract

**Simple Summary:**

African swine fever (ASF) is one of the most important animal diseases affecting the domestic swine population globally. Whole-genome sequence analysis on the circulating African swine fever virus (ASFV) strains would provide valuable information in tracking the outbreaks of the disease. The aim of this study was to prepare a curated dataset of ASFV genome sequences and investigate genome-wide diversity of circulating ASFV strains. We prepared a curated dataset containing 123 high-quality ASFV genome sequences representing 10 genotypes collected from 28 countries between 1949 and 2020. Phylogenetic analysis based on whole-genome sequences provided high-resolution topology in genotyping ASFV isolates, which was supported by pairwise genome sequence similarity comparison. Wide distribution and high variation of tandem repeat sequences were found in ASFV genomes. Structural variation and highly variable poly G or poly C tracts were also identified. This study improved our understanding on the patterns of genetic variation of ASFV and facilitated future studies on ASFV molecular epidemiology.

**Abstract:**

African swine fever (ASF) is a lethal contagious viral disease of domestic pigs and wild boars caused by the African swine fever virus (ASFV). The pandemic spread of ASF has had serious effects on the global pig industry. Virus genome sequencing and comparison play an important role in tracking the outbreaks of the disease and tracing the transmission of the virus. Although more than 140 ASFV genome sequences have been deposited in the public databases, the genome-wide diversity of ASFV remains unclear. Here we prepared a curated dataset of ASFV genome sequences by filtering genomes with sequencing errors as well as duplicated genomes. A total of 123 ASFV genome sequences were included in the dataset, representing 10 genotypes collected between 1949 and 2020. Phylogenetic analysis based on whole-genome sequences provided high-resolution topology in differentiating closely related ASFV isolates, and drew new clues in the classification of some ASFV isolates. Genome-wide diversity of ASFV genomes was explored by pairwise sequence similarity comparison and ORF distribution comparison. Tandem repeat sequences were found widely distributed and highly varied in ASFV genomes. Structural variation and highly variable poly G or poly C tracts also contributed to the genome diversity. This study expanded our knowledge on the patterns of genetic diversity and evolution of ASFV, and provided valuable information for diagnosis improvement and vaccine development.

## 1. Introduction

African swine fever (ASF) is a lethal, contagious viral disease of domestic pigs and wild boars caused by African swine fever virus. ASF also affects African wild suits (warthogs and bushpigs) in an asymptomatic carrier state. Soft ticks serve as a natural reservoir and transmit the disease to suits. First identified in Kenya in 1921, ASF was endemic in most countries in Africa, Europe, and Asia, and was also reported in Dominica and Haiti [1,2]. With no vaccine or treatment available, affected pigs were culled in order to try to contain the outbreaks. More than 50 countries are now affected by ASF, causing the death or culling of more than nine million pigs [3]. The pandemic spread of ASF has had serious effects on the global pig industry, even leading to a critical global heparin shortage [4,5,6].

ASFV is a large, enveloped DNA virus, member of the family *Asfarviridae* [7]. The genome of ASFV is a linear double-stranded DNA (dsDNA) molecule with a length of 171 kb to 193 kb with terminal inverted repeats and hairpin loops [8]. It has a conserved central region (CCR) of about 125 kb, containing genes involving virus replication, assembly, and host cell function modulation. The left variable region and the right variable region are variable in size, containing different numbers of five multigene family (MGF) genes: MGF 100, 110, 300, 360, and 505/530.

Phylogenetic analysis based on the 440 bp-length partial sequence of the approximately 1500 bp-length B646L gene has revealed 24 genotypes of ASFV [9]. All of the 24 different genotypes of ASFV were found to be endemic in Africa. In the 1950s, ASFV genotype I invaded into Portugal and spread to other countries in Europe until it was eradicated in the mid-1990s, with the exception of Sardinia, Italy [10]. Due to this expansion, the disease was established in Sardinia, Italy. In 2007, ASFV genotype II was introduced into Georgia and swept across most of Europe. In 2018, ASFV genotype II was reported in China and other Asian countries [11,12]. In order to distinguish the closely related strains in the same genotype, several other viral genes (E183L, B602L, KP86R, I196L, and EP402R) and IGRs (J286L, BtSj, I73R/I329R, and I78R/I215L) have been used as molecular markers [13,14,15]. However, the information provided by these markers is limited and could not be interpreted unambiguously to trace the origin of closely related virus strains.

The development of next-generation sequencing (NGS) and third-generation sequencing techniques enabled the reduction of genome sequencing cost, and hence made large-scale sequencing for large dsDNA virus genomes feasible [16,17]. To date, more than 140 ASFV genome sequences of different origins and variable virulence, mainly belonging to genotype I and genotype II, have been determined and deposited in GenBank [18,19,20,21,22]. The increased number of full genome sequences of ASFV strains enabled more robust and more complex phylogenetic analyses [23,24,25]. However, the quality of some ASFV genome sequences in the public database was not high enough for detailed analyses [26]. It is, therefore, very important to prepare a curated dataset of high-quality ASFV genome sequences for further genomic characterization and phylogenetic analyses. In this study, we prepared a curated dataset of publicly available high-quality ASFV genome sequences. We further conducted an in-depth analysis on the genomic diversity of ASFV genome sequences based on the largest up to date dataset.

## 2. Materials and Methods

### 2.1. Published ASFV Genome Sequences Acquisition

All published ASFV complete genomes were retrieved from the NCBI Nucleotide database on 10 November 2021. Source information including genotype, country, host, sampling location, and collection date were obtained either by GenBank records or related publications.

### 2.2. Sequence Alignment

The ASFV genome sequences were aligned using MAFFT software (version 7.475) [27]. Genome sequences containing artificial modification, annexation bases, unknown bases, and unconfirmed large-fragment deletion were identified.

### 2.3. Maximum Likelihood Phylogenetic Analysis

Maximum likelihood (ML) phylogenetic trees were estimated by RAxML (version v8.2.12) [28] using the GTR-GAMMA nucleotide substitution model. ML bootstrapping was performed with 1000 replicates in order to assess the robustness of tree topologies. The final tree was midpoint rooted by FigTree v1.4.2.

### 2.4. Sequence Diversity Analysis

Pairwise sequence distance was inferred by the maximum-likelihood method from the nucleotide alignment of genome sequences using MEGA version 10.2.6 software, assuming a TN93 model of base substitution (equal substitution rates among sites and between transitional and transversional substitutions) [29]. A heatmap was drawn on the genome sequence identity using pheatmap packages (version 1.0.12).

### 2.5. ORF Analysis

ORF in each genome sequence was identified using GATU, setting the minimal threshold for the length of ORF as 30 codons [30]. Using Georgia 2007/1 as the comparator, the sequence identity of each putative ORF of each strain was compared to its Georgia 2007/1 ortholog. A custom script was used to show the distribution of ORF along the genome.

### 2.6. Tandem Repeat Sequences Analysis

Tandem repeat sequences (TRS) across the genome were identified using the TRF program (version 4.09) [31]. The match, mismatch, and delta parameters were selected as 2, 7, and 7, and the minimum score was set to 50.

## 3. Results

### 3.1. Curated ASFV Complete Genome Sequences Dataset

A total of 147 published ASFV complete genomes were retrieved from the NCBI Nucleotide database on 10 November 2021. Source information including genotype, host, sampling location, and collection date was obtained either by GenBank records or a search of the literature. The genomes in the dataset belonged to 11 genotypes including genotype I (73 genomes), genotype II (52), genotype III (2), genotype IV (2), genotype V (1), genotype VII (1), genotype VIII (1), genotype IX (6), genotype X (5), genotype XX (3), and genotype XXII (1).

All of the genome sequences were aligned by MAFFT software. Mutations in each genome sequence were checked manually. Artificial modifications of the codons in the open reading frame in the reverse strand of the genome were found in 6 genomes collected from South Africa (MN394630.3, MN641876.2, MN641877.2, and MN336500.3), Zambia (MN318203.3), and Zaire (MN630494.2), as partly shown in Figure S1. A genome from Armenia (LR881473.1) had extremely low sequence homology to other genotype II genomes and was found to be a contamination of a genotype I strain [32]. Low genome coverage or sequence similarity was found in 4 ASFV genotype II genomes collected from Viet Nam (MW465755.1, MT180393.1, MT166692.1) and China (MW361944.1) (Table S1). Annexation bases or expand unknown bases were found in 2 sequences collected in Georgia (MH910495.1 and MH910496.1) and 6 genomes collected in Italy (MW788405.1, MW788407.1-MW788411.1). Unconfirmed large-fragment deletion was found in a sequence collected in China (MH766894.2). The origin of sequences MZ945537 and MZ945536 was unclear. Sequence MK333181.1 was completely identical to sequence MK333180.1. Sequence MN393477.1 was completely identical to sequence MN393476.1. In order to obtain a precise overview of the genome diversity of ASFV strains, these 24 genomes were not included in the dataset (Table S2).

**Figure 1:**
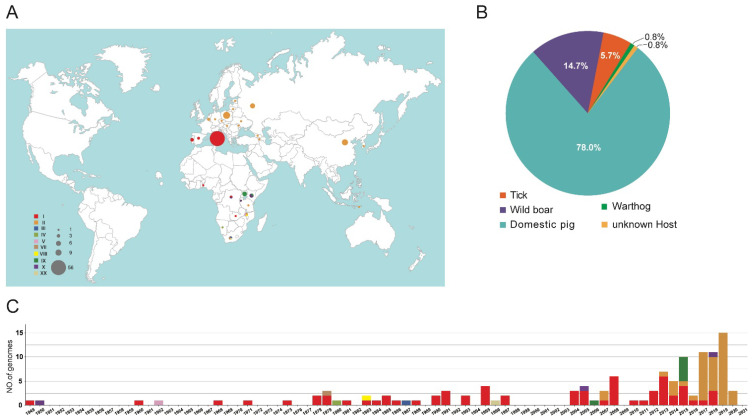
Distribution of African swine fever virus genome sequences included in the curated dataset. (**A**) Map indicates the collection locations of the ASFV genome sequences. (**B**) Distribution of the host of the ASFV genome sequences. (**C**) Distribution of the collection date of the ASFV genome sequences.

The final dataset included a total of 123 ASFV genome sequences, belonging to 10 genotypes: genotype I (64 genomes), II (42), III (1), IV (1), V (1), VII (1), VIII (1), IX (6), X (5), and XX (1) (Table S3). These genomes were collected in domestic pigs (96 genomes), wild boars (18), ticks (7), warthog (1) or unknown host (1) from Italy (56 genomes), China (9), Poland (12), Russia (6), 10 countries in Africa (22 genomes), 4 other countries in Asia (4 genomes), and 10 other countries in Europe (14 genomes) from 1949 to 2020 (Figure 1). As shown in Figure 1, African countries are significantly underrepresented. The majority of the sequences were collected during the last ten years.

### 3.2. Phylogenetic Reconstruction

**Figure 2:**
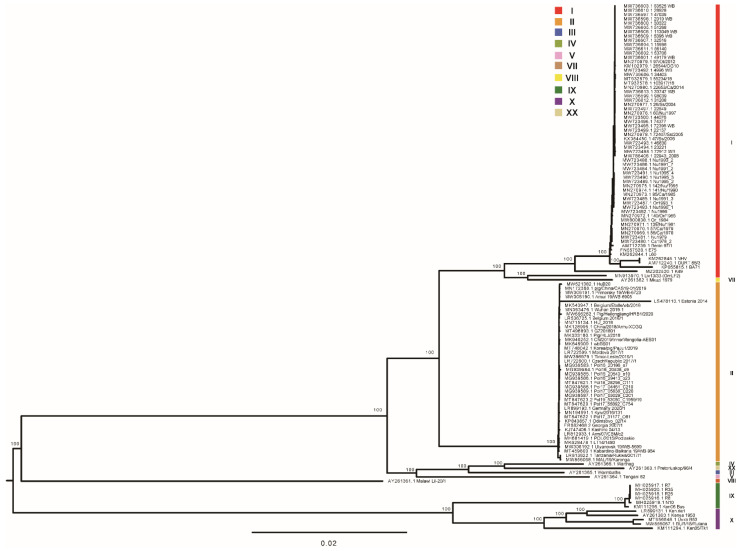
Maximum likelihood phylogenetic tree of ASFV genome sequences in the curated dataset. The tree is midpoint rooted. The scale bar is given in numbers of substitutions per site. Bootstrap resampling (1000 replications) support values are shown at the nodes.

The maximum likelihood phylogenetic dendrogram of ASFV was constructed from the alignment of genome sequences. Two primary clades were found with 100% bootstrap support (Figure 2). Primary clade I included two subclades, comprising previously identified genotype X and genotype IX isolates, respectively, which were collected from Kenya, Congo, and Uganda. Primary clade II included three subclades and one singleton (Lil-20 Malawi 1983). One subclade comprised distinctive isolates of genotype V, III, IV, and XX collected from Malawi, Zambia, Namibia, and South Africa. The second subclade was composed of all of the genotype II strains collected in Africa, Europe, and Asia. The third subclade was comprised of a cluster of Mkuzi 1979 and Liv13/33 (OmLF2), a singleton of K49, and a cluster of all of the other genotype I isolates. It is of note that Liv13/33 (OmLF2), a strain previously classified into genotype I by p72 genotyping, was found to be closely related to Mkuzi 1979, a genotype VII strain. K49, which was previously identified as a genotype I isolate, was found remotely related to all of the other genotype I strains. It has been revealed that phylogenetic analysis based on high-quality whole-genome sequences could provide high-resolution genotyping for ASFV.

### 3.3. Genome-Wide Sequence Similarity

**Figure 3:**
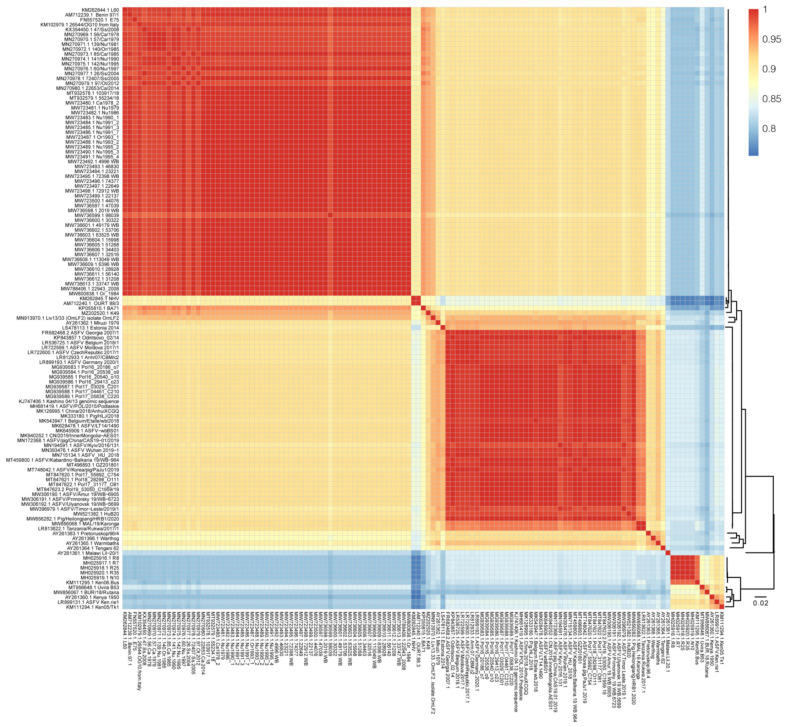
Sequence similarity matrix plot of ASFV genome sequences in the curated dataset. The level of identity of pairwise genome sequences is indicated by different colors. Dark red represents 100% identity, and blue represents lower identity. The maximum likelihood phylogenetic tree of ASFV genome sequences is shown on the right.

Pairwise sequence similarity was calculated to investigate the genome-wide diversity of ASFV. The overall whole-genome sequence similarity varied between 75.4% and 99.9% (Figure 3). Inter-genotype similarity ranged between 75.4% and 94.7%. The lowest inter-genotype pairwise similarity was found between genotype I and genotype X (Kenya 1950), between 75.4–83.4%. The highest inter-genotype pairwise similarity reached 82.4–94.7%, between genotype I and genotype II. Within genotype II, the sequence divergence ranged between 92.4% and 99.9%. Within genotype I, the pairwise sequence similarity diverged from 85.4% to 99.9%. It is of note that a drop in sequence similarity (85.4–89.9%) was found between NH/P68-like strains and other genotype I strains, which was caused by large fragment sequence deletion in these strains. Mkuzi 1979, Liv13/33 (OmLF2), and K49 shared 85.4–88.1% with the NH/P68-like strains and 89.7–95.4% sequence similarity with other genotype I strains. All of the other genotype I strains shared a high similarity, between 94.8% and 99.9%. It is suggested that the relationship between Mkuzi 1979, Liv13/33 (OmLF2), and K49 and genotype I strains should be reconsidered from the perspective of whole-genome sequence similarity.

### 3.4. Genome Annotation

In order to investigate the open reading frame (ORF) distribution in ASFV genomes, gene annotation information was obtained by GenBank records. For genomes with no gene annotation information available, ORFs were identified using GATU software. The length of the genome, coding region sequence (CRS), and CCR are listed in Table S4. The length of the genomes ranged from 171,235 bp to 193,886 bp. The length of the CRS of each genome ranged from 171,046 bp to 192,664 bp. The length of CRS differed between different genotypes, which ranged from 171,046 bp to 186,915 bp in genotype I and from 181,232 bp to 189,797 bp in genotype II. The length of the CCR of each genome was identical, with minor diversity from 129,288 bp to 132,794 bp. No significant difference was observed in the length of CCR in different genotypes.

The ORF distribution in the representative strains of 10 different ASFV genotypes was compared (Figure S2). Gain or loss of predicted ORF was observed mainly in the LVR and RVR regions, especially in members of MGF. As shown in Table 1, the number of MGF110 members ranged between 6 and 11 in different genotypes. The number of MGF360 members varied between 10 and 14 in LVR, and between 3 and 5 in RVR. The number of MGF505 members ranged between 8 and 9 in LVR. The sequence identity of each ORF to that of the reference strain Georgia 2007/1 is also shown in Figure S2. It is of interest that several genes located in the CCR showed a considerable sequence variation, including A118R, A238L, EP153R, EP402R, and E66L.

### 3.5. Structural Variation

**Figure 4:**
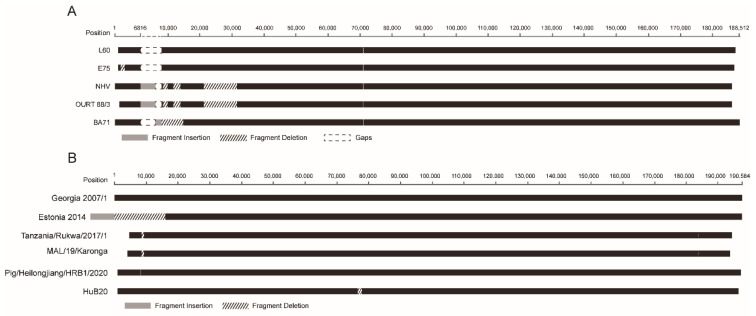
Structural variations in ASFV genotype I (**A**) and genotype II (**B**) genomes. Fragment insertion is shown in dark grey blocks. Fragment deletion is shown in dashed blank blocks. Blank blocks indicate gaps introduced for alignment purposes. Nucleotide position in the reference genome L60 (**A**) and genome Georgia 2007/1 (**B**) is shown.

Structural variations include deletion and insertion of sequence spanning at least 50 base pairs. Structural variations occurred during the circulation of ASFV in the host or passage of the virus in cell cultures. With the curated dataset, we investigated the structural variations of ASFV genomes in genotype I and genotype II. Structural variations were found in five ASFV genotype I genomes, including BA71 (one deletion/insertion), E75 (one deletion), L60 (one deletion), NH/P68 (three deletions and one insertion), and OURT 88/3 (same as NH/P68). The detail is shown in Figure 4A. Structural variations were also found in five ASFV genotype II genomes, including Tanzania/Rukwa/2017/1 (three deletions), MAL/19/Karonga (three deletions), Estonia 2014 (one deletion/insertion), Pig/Heilongjiang/HRB1/2020 (one recombination), and HuB20 (one deletion). The distribution of the structural variations in these genomes is shown in Figure 4B.

### 3.6. Tandem Repeat Sequences Variation

Tandem repeat sequences (TRS) across the genomes of the ten representative strains of each genotype were identified using the TRF program. In each genome, approximately 30 TRS regions were identified. The distribution of TRSs in the ASFV genome is shown in Figure S2. Fourteen TRSs shared by all the ten representative strains were further investigated (Table 2). Seven TRSs were located in the coding region of EP402R, C84L, B475L, B602L, B407L, B183L and I196L. The other seven TRSs were located in the non-coding region between two ORFs. Five TRSs were found to be compound TRSs, which were composed of two or three single TRSs. The length of repeat units ranged from 3 to 66 bp, the majority ranging from 10 to 20 bp. The copy number of repeat units in each TRS varied considerably among different genotypes. For instance, for the TRS located in the non-coding region between MGF505-9R and MGF505-10R, the 17-bp repeat unit (GTTCAGTTAAGACAGTA or GTTAAGACAATAGTTTT) had 7 copies in Ken06 (KM111295.1) but 32 copies in Malawi Lil-20/1 (AY261361.1). In the coding region of the B602L gene, a TRS with a 12-bp repeat unit (GTGCTTGTACAA) was identified in the 487-nucleotide site at the 5’ end of the 1593-bp ORF, which is also known as a central variable region (CVR). In the CVR, the copy number of the repeat unit ranged variably from 7 in the strain Tengani 62 (genotype V) to 31 in the strain Malawi Lil-20/1 (genotype VIII). Mutations in each repeat unit were also observed, including single nucleotide polymorphisms and indels.

**Figure 5:**
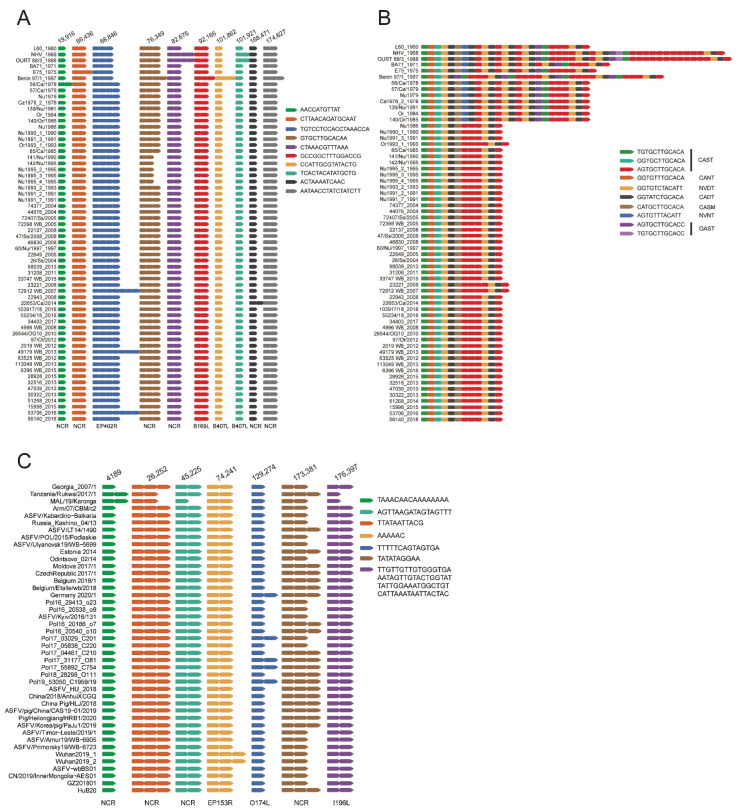
Sequence variation of the tandem repeat sequences (TRS) in ASFV genomes. (**A**) Variation of TRS in other regions in ASFV genotype I genomes. (**B**) Variation of TRS in the coding region of B602L gene in ASFV genotype I genomes. (**C**) Variation of TRS in ASFV genotype II genomes. The arrangement of the TRS in each genome was listed according to the nucleotide sequence. Each arrow of a different color represents a type of repeat unit with a specific nucleotide sequence. The nucleotide sequence of each type of repeat unit was also shown. The amino acid sequence of each type of repeat unit in the TRS in the coding region of B602L gene was also shown.

In the coding region of different ASFV genotype I strains, variation in the number of repeat units was found in 11 TRS. Six TRS variations were located in the non-coding region, whereas five TRSs were located in the coding region of EP402R, B169L, B407L, and B602L (Figure 5A,B). The most variable TRS was found in CVR (B602L), where variation in the number and sequence of the 12-bp repeat unit was identified. In CVR, according to the nucleotide sequence in each repeat unit, 10 different repeat units were identified corresponding to seven types of amino acid sequence. Based on the number and arrangement of the repeat units in CVR, ASFV genotype I strains could be classified into four types. Benin 97 had a unique arrangement of 36 repeat units. NHV and OURT 88/3 shared an array of 45–46 repeat units. L60, BA71, E75, and strains collected from Sardinia from 1978 to 1985 showed a similar line of 25–29 repeat units. All of the other strains collected from Sardinia from 1986 to 2018 had the same queue of 12–13 repeat units. In conclusion, CVR variation could be used to differentiate historical ASFV genotype I strains, but could not differentiate contemporary strains.

TRS variation was further investigated in ASFV genotype II genomes. Variation in the number of repeat units was found in seven TRSs in the coding region of different genotype II ASFV strains (Figure 5C). The most variable TRS was found in I73R/I329L IGR. Based on the number of repeat units in I73R/I329L IGR-TRS, the genome-sequenced genotype II ASFV strains in the dataset could be classified into 2 types.

### 3.7. Highly Variable Poly G or Poly C Tracts

**Figure 6:**
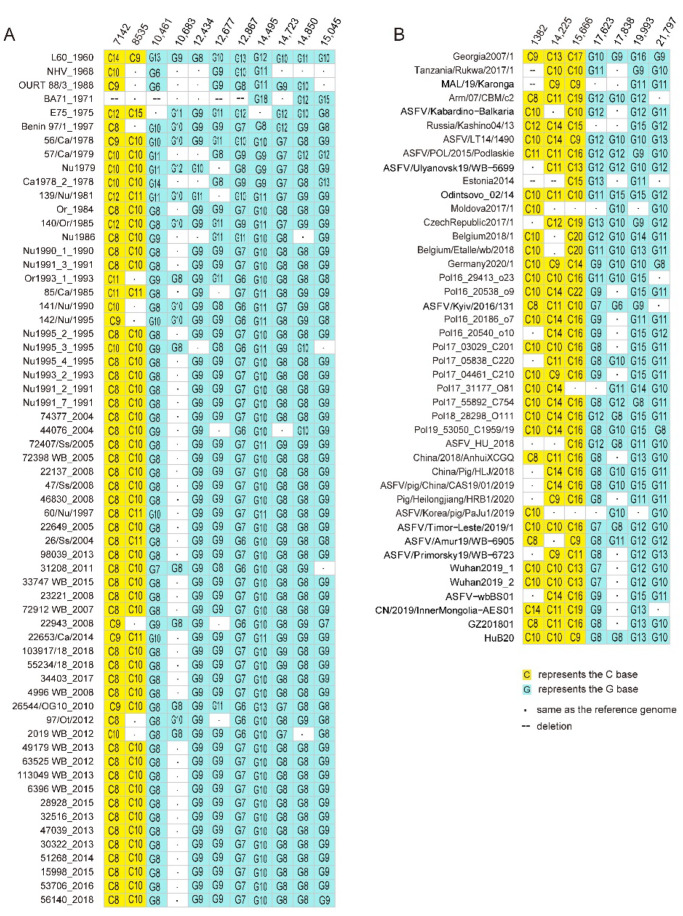
Sequence variation of the highly variable poly G or poly C repeats sequence in ASFV genotype I (**A**) and genotype II (**B**) genomes. Nucleotide position in the reference genome L60 (**A**) and genome Georgia 2007/1 (**B**) is shown.

Highly variable numbers of G or C repeats in ASFV genome have been occasionally reported. In this study, highly variable poly G or C repeats distribution in genotype I and genotype II were investigated. Highly variable numbers of G or C repeats were observed from the alignment of ASFV genotype I strains at 11 poly G or poly C regions at nucleotide position 7142 (poly C_8-14_), 8535 (poly C_9-15_), 10,461 (poly C_8-13_), 10,683 (poly G_8-12_), 12,434 (poly G_8-11_), 12,677 (poly G_8-11_), 12,867 (poly G_6-13_), 14,495 (poly G_8-13_), 14,723 (poly G_7-12_), 14,850 (poly G_8-12_), and 15,045 (poly G_8-15_) of the genome of reference strain L60 (KM262844.1) (Figure 6A). Two sites were located in the open reading frame (ORF) of MGF-110-5L (nt7142) and MGF-110-13L (nt8535), respectively. The C variance might change the number of glycine repeats in the polyglycine site or provide frame-shift modulation on the products of these genes, causing an extension of MGF-110-5L (16 aa or 154 aa longer), or MGF-110-13L (29 aa or 154 aa longer).

From the alignment of genotype II ASFV strains, seven highly variable poly G or poly C regions were identified at nucleotide position 1382 (poly C_8-14_), 14,225 (poly C_9-14_), 15,666 (poly C_9-22_), 17,623 (poly G_7-13_), 17,838 (poly G_6-15_), 19,993 (poly G_8-16_), and 21,797 (poly G_8-13_) of the genome of reference strain Georgia 2007/1(Figure 6B). Most of these highly variable poly G/C sequences were located in the non-coding region. Two sites were located in the coding region of MGF-110-14L (nt 14,224) and MGF-110-13L (ref nt 15,665), respectively. The C variance might change the number of glycine repeats in the polyglycine site or provide frame-shift modulation on the products of these genes, causing extension of MGF-110-14L (6 aa or 117 aa long), or truncation of MGF-110-13L (145 aa or 146 aa short).

## 4. Discussion

Genome characterization of circulating ASFV strains could expand our knowledge on the genetic diversity and evolution of ASFV, providing valuable information for diagnosis improvement and vaccine development. The value of this kind of study depends on the quality of the genome sequences. Although more than 140 genome sequences have been published, approximately 10% of the genomes are not precisely determined. It is highly suggested that precautions should be taken to produce high-quality ASFV genome sequences [26]. Several deep-sequencing workflows have been developed for the fast and efficient generation of high-quality ASFV whole-genome sequences by using either next-generation sequencing alone or a combination of next-generation sequencing and third-generation sequencing [16,19,33,34,35]. No matter which workflow is taken, it is important that the genome assembly generated from the workflow should be checked manually. Sequence variations that are different from the reference sequence should be supported by a high depth of reads. Annexation bases or unknown bases in the sequences should be checked, corrected, or confirmed by Sanger sequencing, as well as fragment deletion. These steps would significantly improve the quality of the genome sequences and make the best use of the great efforts which have been made to obtain the genome sequences.

The curated dataset was used to reconstruct the phylogenetic tree of ASFV genome sequences. A robust phylogenetic tree was obtained. All of the major branches were well-supported. Our study showed new insight into the genotyping of Mkuzi 1979, Liv13/33, and K49. By p72 genotyping, Mkuzi 1979 was classified with RSA/1/98 (AF302818) into genotype VII [36,37]. Liv13/33 was classified into genotype I by p72 genotyping [38]. K49 was classified into genotype I according to the description of the sequence submitter. In this study, it is supported by strong evidence that Liv13/33 and Mkuzi 1979 grouped into a distinct cluster, separating from the cluster of genotype I strains. It is therefore suggested that Liv13/33 and Mkuzi 1979 should be classified into genotype VII. In our study, K49 formed a singleton which was closely related to but separate from the cluster of genotype I or genotype VII strains. Achieving more genome sequences of genotype VII and genotype I strains collected in Africa would help to settle this problem.

To date, a total of 24 genotypes of ASFV have been identified worldwide. However, genome sequences of only 10 genotypes were available, with genomes of genotype I and genotype II collected in Eurasia overrepresented. The lack of ASFV genome sequences of the remaining 14 genotypes dramatically limited the breadth and depth of phylogenetic analysis of ASFV currently [39]. More resources should be allocated to generate more whole-genome sequences for historical and contemporary ASFV strains collected in Africa, belonging to all 24 genotypes.

Variation of tandem repeat sequences, especially CVR region and IGR I73R/I329R, has been reported and used to enhance discrimination of ASFV isolates [13,40,41,42]. However, the genome-wide distribution of TRSs in ASFV has never been reported. According to our result, TRSs were widely distributed in the ASFV genomes. Some TRSs identified in this study have not been previously reported. The high quality of genome sequences in genotype I and genotype II enabled the in-depth investigation into the TRS variation during the circulation of ASFV. Although several TRS variations were identified in genotype I and genotype II, respectively, only one TRS in the NCR of MGF 360-9L and 10L was found simultaneously varied in the genome-sequence-available genotype I and II strains. Although dramatic variation in CVR was identified in different genotype I strains, no variation was found in this region in genotype II strains included in this study. However, a CVR variant was reported in ASFV strains in wild boar from a limited area in southern Estonia in 2015 and 2016 [43]. The role of TRS variation in discriminating closely related ASFV strains within a genotype needs to be further investigated.

Variation in stretches of poly G or poly C region in ASFV have been previously reported [18]. In this study, for the first time, we listed all of the highly variable poly G or poly C tracts observed in the genomes of ASFV genotype I and genotype II strains, respectively. It has been revealed that these regions are not only highly variable, but also irregular. One explanation is that this is caused by sequencing errors because the current sequencing methods reach their limit in determining the long homopolymer G or C stretches [26]. The other possibility is that it represents the intrastrain sequence variation due to replication slippage on the homopolymeric tracts [44].

## 5. Conclusions

In conclusion, we prepared a curated dataset of ASFV full-length genome sequences for further studies on genome characterization for new outbreaks and genomic epidemiology analysis for ASFV. Our whole-genome-wide diversity analysis based on the curated dataset improved our understanding of the evolution of ASFV during circulation, and thus might help control the spread of this important animal disease. The curated dataset described here will be updated regularly to include the newly published ASFV full genome sequences.

## Figures and Tables

**Table 1:** Number of MGF members present in the LVR and RVR of different genotypes of ASFV genomes.

Genotype	LVR					RVR		
	MGF 100	MGF 110	MGF 300	MGF 360	MGF 505/530	MGF 100	MGF 360	MGF 505/530
I (KM262844.1)	0	6	3	13	9	2	3	1
II (FR682468.2)	1	11	3	14	9	2	5	1
III (AY261365.1)	1	11	3	13	9	2	4	1
IV (AY261366.1)	1	9	3	11	9	2	4	1
V (AY261364.1)	1	6	3	12	9	2	4	1
VII (AY261362.1)	1	10	3	13	9	2	4	1
VIII (AY261361.1)	1	10	3	12	8	2	3	1
IX (KM111295.1)	1	9	3	10	8	2	3	1
X (AY261360.1)	1	11	3	14	8	2	3	1
XX (AY261363.1)	1	9	3	13	9	2	4	1

**Table 2:** Distribution of 14 common tandem repeat sequences (TRS) in the ASFV genome.

TRS	Site	Start	Stop	Pattern	RU	Sequence	Length	Number
TRS1	NCR MGF505-9R_10R	45,221	45,491	(A)n	A	GTTCAGTTAAGACAGTA/GTTAAGACAATAGTTTT	17	7–32
TRS2	EP402R	75,257	75,322	(A)n	A	AAACCATGTCCTCCACCC	18	7–14
TRS3	C84L	82,690	82,785	(A)n	A	GTGCCTGCACAA	12	3–8
TRS4	NCR C84L_C717R	82,976	83,053	(A)n	A	GTTTTAGCTT *	10	2–8
TRS5	NCR C122R_C257L	85,722	85,898	(A)n-X-(B)n	A	CAAGTATTTTCTATAGC	17	3–7
					B	CATTAAAACAAAGAGTCTAATAAGACGCTTTAATGGG *	37	1–6
TRS6	NCR C315R_C147L	89,123	89,187	(A)n	A	CTAAACGTTTAAA *	13	3–6
TRS7	B475L	99,197	99,397	(A)n-(B)n	A	CAAGGATATTTTCTTTGATAT	21	0–4
					B	CAAGAATATTTTCTTCTTCAGGTTTATCCTGACCAAACT *	39	3–5
TRS8	B602L	102,670	102,789	(A)n	A	GTGCTTGTACAA	12	8–31
TRS9	B407L	108,045	108,239	(A)n	A	CCTCGGCGCGTCTTA	15	10–14
TRS10	E183L	163,354	163,485	(A)n-X-(B)n	A	GCGGCCGCAGGT	12	0–7
					B	GGGTTGTCCGTAACT	15	3–6
TRS11	NCR E146L_E199L	166,659	166,816	(A)n	A	CTTAAGTTTATTGCTCATGG *	20	2–27
TRS12	NCR I73R_I329L	173,344	173,411	(A)n-(B)n	A	TAAATGTAGAATAACACAGTTAAGCAATAAATAACAAG	38	1–8
					B	TATATAGGAA	10	3–17
TRS13	NCR I329L_I215L	174,667	174,878	(A)n-X-(B)n-X-(C)n	A	CTAAATTCTAAGCA	14	3–21
					B	CAT	3	11–20
					C	TCTTCA	5	3–5
TRS14	I196L	176,397	176,528	(A)n	A	TTGTTGTTGTGGGTGAAATAGTTGTACTGGTATTATTGGAAATGGCTGTCATTAAATAATTACTAC *	66	1–3

Note: RU: repeat unit, * high variation in sequence and number of RU in the TRS.

## Data Availability

All the data were downloaded from https://www.ncbi.nlm.nih.gov/nuccore (accessed on 10 September 2022).

## Supplementary Materials

The following supporting information can be downloaded at: https://www.mdpi.com/article/10.3390/ani12182446/s1, Figure S1: Artificial modifications of the codons in the open reading frame (ORF) in the reverse strand of 6 genomes: MN394630.3, MN641876.2, MN641877.2, MN336500.3, MN318203.3 and MN630494.2. Part of the ORF was marked from the genome sequence alignment. Figure S2: The distribution of ORF and TRS in the representative strains of 10 different ASFV genotypes. The sequence identity of each ORF to that of the reference strain Georgia 2007/1 was also shown. Table S1: Low genome coverage or sequence similarity in 4 ASFV genotype II genomes. Table S2: ASFV genome sequences not used in the curated dataset. Table S3: A curated dataset of ASFV genome sequences. Table S4: The length of genome, coding region sequence (CRS) and conserved central region (CCR) of the ASFV genome sequences in the curated dataset. References [45,46,47,48,49,50,51,52,53,54,55,56,57,58,59,60,61,62,63,64,65] are cited in the supplementary materials.
