# Supplementary material for: Genome-Wide Diversity Analysis of African Swine Fever Virus Based on a Curated Dataset
Source: Animals (Basel). 2022 Sep 16;12(18):2446. doi: 10.3390/ani12182446 (PMC9495133; doi:10.3390/ani12182446)
Supplement: Supplementary file 1 [file animals-12-02446-s001.zip › Supplemental figure legends.pdf]

Figure S1. Artificial modifications of the codons in the open reading frame (ORF) in the reverse strand of 6 genomes: MN394630.3, MN641876.2, MN641877.2, MN336500.3, MN318203.3 and MN630494.2. Part of the ORF was marked from the genome sequence alignment.

Figure S2. The distribution of ORF and TRS in the representative strains of 10 different ASFV genotypes. The sequence identity of each ORF to that of the reference strain Georgia 2007/1 was also shown.
