# Supplementary material for: Genome-Wide Diversity Analysis of African Swine Fever Virus Based on a Curated Dataset
Source: Animals (Basel). 2022 Sep 16;12(18):2446. doi: 10.3390/ani12182446 (PMC9495133; doi:10.3390/ani12182446)
Supplement: Supplementary file 1 [file animals-12-02446-s001.zip › Supplemental TablesV2.pdf]

Table S1. Low genome coverage or sequence similarity in 4 ASFV genotype II genomes.

**Sequence**	**Length**	**Coverag**	**Similarity**
FR682468.2	190,584	100%	100%
MT180393.1	186,496	97.85%	97.80%
MT166692.1	166,863	87.56%	87.50%
MW361944.1	186,471	97.84%	69.00%
MW465755.1	186,237	97.72%	30.70%

Table S2. ASFV genome sequences not used in the curated dataset.

NCBI Acc No	Strain	Host	Length	Note	Collection date	Country	Genotype	Ref
MN318203.3	LIV_5_40	Tick	183,291	Artificial modification	1983	Zambia	I	[45]
MN394630.3	SPEC_57	Tick	186,118	Artificial modification	1985	South Africa	III	[45]
MN641876.2	RSA_W1_1999	warthog	187,621	Artificial modification	1999	South Africa	IV	[45]
MN641877.2	RSA_2_2004	Wild boar	189,903	Artificial modification	2004	South Africa	XX	[45]
MN630494.2	Zaire	Pig	184,820	Artificial modification	1977	Zaire	XX	[45]
MN336500.3	RSA_2_2008	Tick	190,242	Artificial modification	2008	South Africa	XXII	[45]
LR881473.1	Arm/07/CBM/c4	Pig	192,206	Contamination	2007	Arminia	II	[32]
MW465755.1	VNUA-ASFV-05L1/HaNam/VN/2020	Pig	186,237	30.7% similarity	2020	Viet_Nam	II	NA
MT180393.1	ASFV_NgheAn_2019	Pig	186,498	97.85% genome coverage	2019	Viet_Nam	II	NA
MT166692.1	ASFV_Hanoi_2019	Pig	166,931	87.56% genome coverage	2019	Viet_Nam	II	NA
MW361944.1	China/GD/2019	Pig	186,471	69% similarity	2019	China	II	NA
MH910495.1	Georgia 2008/1	Pig	189,465	Annexation bases	2008	Georgia	II	[46]
MH910496.1	Georgia 2008/2	Pig	189,315	Expand Ns	2008	Georgia	II	[46]
MW788405.1	1537 WB	Pig	181,697	Expand Ns	2008	Italy	I	[23]
MW788407.1	31479_2005	Pig	181,642	Expand Ns	2005	Italy	I	[23]
MW788408.1	35479_2014	Pig	181,596	Expand Ns	2014	Italy	I	[23]
MW788409.1	SS_1981	Pig	181,735	Expand Ns	1981	Italy	I	[23]
MW788410.1	25185_2008	Pig	181,693	Expand Ns	2008	Italy	I	[23]
MW788411.1	24225_2002	Pig	181,383	Expand Ns	2002	Italy	I	[23]
MH766894.2	ASFV-SY18	Pig	189,354	Unconfirmed deletion	2018	China	II	NA
MK333181.1	DB/LN/2018	Pig	189,404	Same as MK333180.1	2018	China	II	[47]
MN393477.1	Wuhan 2019-2	Pig	190,576	Same as MN393476.1	2019/8/19	China	II	NA
MZ945536.1	Pig/HeN/ZZ-P1/2021	Pig	171,235	Homologious to KM262845.1	2021/3/31	China	I	[48]
MZ945537.1	Pig/SD/DY-I/2021	Pig	172,025	Homologious to KM262845.1	2021/4/27	China	I	[48]

Table S3. A curated dataset of ASFV genome sequences.

NCBI Acc No	Strain	Host	Length	collection date	Country	Genotype	Ref
AM712239.1	Benin 97/1	Domestic pig	182,284	1997	Benin	I	[22]
KM102979.1	26544/OG10	Domestic pig	182,906	2010	Italy	I	NA
MT932578.1	103917/18	Domestic pig	181,759	2018	Italy	I	[49]
MT932579.1	55234/18	Domestic pig	181,761	2018	Italy	I	[49]
MN270969.1	56/Ca/1978	Domestic pig	183,639	1978	Italy	I	[24]
MN270970.1	57/Ca/1979	Domestic pig	183,639	1979	Italy	I	[24]
MN270973.1	85/Ca/1985	Domestic pig	181,816	1985	Italy	I	[24]
MN270980.1	22653/Ca/2014	Domestic pig	181,869	2014	Italy	I	[24]
MN270971.1	139/Nu/1981	Domestic pig	183,645	1981	Italy	I	[24]
MN270974.1	141/Nu/1990	Domestic pig	183,720	1990	Italy	I	[24]
MN270975.1	142/Nu/1995	Domestic pig	183,724	1995	Italy	I	[24]
MN270976.1	60/Nu/1997	Domestic pig	181,651	1997	Italy	I	[24]
MN270979.1	97/Ot/2012	Domestic pig	184,206	2012	Italy	I	[24]
MN270972.1	140/Or/1985	Domestic pig	183,723	1985	Italy	I	[24]
KX354450.1	47/Ss/2008	Domestic pig	184,638	2008	Italy	I	[50]
MW723480.1	Ca1978_2	Domestic pig	181,925	1978	Italy	I	[23]
MW723481.1	Nu1979	Domestic pig	181,859	1979	Italy	I	[23]
MW723482.1	Nu1986	Domestic pig	181,788	1986	Italy	I	[23]
MW723483.1	Nu1990_1	Domestic pig	181,751	1990	Italy	I	[23]
MW723484.1	Nu1991_2	Domestic pig	181,741	1991	Italy	I	[23]
MW723485.1	Nu1991_3	Domestic pig	181,725	1991	Italy	I	[23]
MW723486.1	Nu1991_7	Domestic pig	181,750	1991	Italy	I	[23]
MW723487.1	Or1993_1	Domestic pig	181,741	1993	Italy	I	[23]
MW723488.1	Nu1993_2	Domestic pig	181,748	1993	Italy	I	[23]
MW723489.1	Nu1995_2	Domestic pig	181,753	1995	Italy	I	[23]
MW723490.1	Nu1995_3	Domestic pig	181,740	1995	Italy	I	[23]
MW723491.1	Nu1995_4	Domestic pig	181,697	1995	Italy	I	[23]
MW723492.1	4996 WB	Domestic pig	181,751	2008	Italy	I	[23]
MW723493.1	46830	Domestic pig	181,746	2008	Italy	I	[23]
MW723494.1	23221	Domestic pig	181,770	2008	Italy	I	[23]
MW723495.1	72398 WB	Domestic pig	181,754	2005	Italy	I	[23]
MW723496.1	74377	Domestic pig	181,753	2004	Italy	I	[23]
MW723497.1	22649	Domestic pig	181,753	2005	Italy	I	[23]
MW723498.1	72912 WB	Domestic pig	181,804	2007	Italy	I	[23]
MW723499.1	22137	Domestic pig	181,751	2008	Italy	I	[23]
MW723500.1	44076	Domestic pig	181,755	2004	Italy	I	[23]
MW736597.1	47039	Domestic pig	47,039	2013	Italy	I	[23]
MW736598.1	2019 WB	Domestic pig	181,742	2012	Italy	I	[23]
MW736599.1	98039	Domestic pig	181,753	2013	Italy	I	[23]
MW736600.1	30322	Domestic pig	181,745	2013	Italy	I	[23]
MW736601.1	49179 WB	Domestic pig	181,793	2013	Italy	I	[23]
MW736602.1	53706	Domestic pig	181,813	2016	Italy	I	[23]
MW736603.1	63525 WB	Domestic pig	181,733	2012	Italy	I	[23]
MW736604.1	15998	Domestic pig	181,761	2015	Italy	I	[23]
MW736605.1	51268	Domestic pig	181,753	2014	Italy	I	[23]
MW736606.1	34403	Domestic pig	181,759	2017	Italy	I	[23]
MW736607.1	32516	Domestic pig	181,758	2013	Italy	I	[23]
MW736608.1	113049 WB	Domestic pig	181,754	2013	Italy	I	[23]
MW736609.1	6396 WB	Domestic pig	181,756	2015	Italy	I	[23]
MW736610.1	28928	Domestic pig	181,738	2015	Italy	I	[23]
MW736611.1	56140	Domestic pig	181,759	2018	Italy	I	[23]
MW736612.1	31208	Domestic pig	181,684	2011	Italy	I	[23]
MW736613.1	33747 WB	Domestic pig	181,753	2015	Italy	I	[23]
MW788406.1	22943_2008	Domestic pig	181,733	2008	Italy	I	[23]
MW800838.1	Or_1984	Domestic pig	181,924	1984	Italy	I	[23]
MN270977.1	26/Ss/2004	Domestic pig	184,581	2004	Italy	I	[24]
MN270978.1	72407/Ss/2005	Domestic pig	181,699	2005	Italy	I	[24]
AM712240.1	OURT 88/3	Tick	171,719	1988	Portugal	I	[22]
KM262844.1	L60	Domestic pig	182,362	1960	Portugal	I	[33]
KM262845.1	NHV	Domestic pig	172,051	1968	Portugal	I	[33]
FN557520.1	E75	Domestic pig	181,187	1975	Spain	I	[36]
KP055815.1	BA71	Domestic pig	180,365	1971	Spain	I	[33]
LR812933.1	Arm/07/CBM/c2	Domestic pig	190,145	2007	Arminia	II	[32]
LR536725.1	Belgium 2018/1	Wild boar	190,599	2018/9	Belgium	II	[18]

MK543947.1	Belgium/Etalle/wb/2018	Wild boar	190,202	2018/9/10	Belgium	II	[51]
MK128995.1	China/2018/AnhuiXCGQ	Domestic pig	189,393	2018/9/2	China	II	[19]
MK333180.1	Pig/HLJ/2018	Domestic pig	189,404	2018/9/5	China	II	[47]
MK645909.1	ASFV-wbBS01	Wild boar	189,394	2018/11/1	China	II	NA
MK940252.1	CN/2019/InnerMongolia-AES01	Domestic pig	189,403	2019/2/19	China	II	NA
MN172368.1	ASFV/pig/China/CAS19-01/2019	Domestic pig	189,405	2019/1/2	China	II	[52]
MN393476.1	Wuhan 2019-1	Domestic pig	190,576	2019/8/19	China	II	NA
MT496893.1	GZ201801	Domestic pig	189,393	2018/12/22	China	II	NA
MW521382.1	HuB20	Domestic pig	188,643	2020/10/1	China	II	NA
MW656282.1	Pig/Heilongjiang/HRB1/2020	Domestic pig	189,355	2020/9/12	China	II	[48]
LR722600.1	CzechRepublic 2017/1	Wild boar	190,594	2017/6	Czech Republic	II	[26]
LS478113.1	Estonia 2014	Wild boar	182,446	2014/9	Estonia	II	[53]
FR682468.2	Georgia 2007/1	Domestic pig	190,584	2007/1/4	Georgia	II	[21]
LR899193.1	Germany 2020/1	Wild boar	190,592	2020/9/10	Germany	II	NA
MN715134.1	ASFV_HU_2018	Wild boar	190,601	2018/4/24	Hungary	II	[35]
MK628478.1	ASFV/LT14/1490	Wild boar	189,399	2014/1	Lithuania	II	[54]
MW856068.1	MAL/19/Karonga	Domestic pig	183,325	2019	Malawi	II	[55]
LR722599.1	Moldova 2017/1	Domestic pig	190,598	2017	Moldova	II	[16]
MG939583.1	Pol16_20186_o7	Domestic pig	189,401	2016	Poland	II	[56]
MG939584.1	Pol16_20538_o9	Domestic pig	189,399	2016	Poland	II	[56]
MG939585.1	Pol16_20540_o10	Domestic pig	189,405	2016	Poland	II	[56]
MG939586.1	Pol16_29413_o23	Domestic pig	189,393	2016	Poland	II	[56]
MG939587.1	Pol17_03029_C201	Wild boar	189,405	2017	Poland	II	[56]
MG939588.1	Pol17_04461_C210	Wild boar	189,401	2017	Poland	II	[56]
MG939589.1	Pol17_05838_C220	Wild boar	189,393	2017	Poland	II	[56]
MH681419.1	ASFV/POL/2015/Podlaskie	Wild boar	189,394	2015/2	Poland	II	[57]
MT847620.1	Pol17_55892_C754	Domestic pig	189,414	2017	Poland	II	[58]
MT847621.1	Pol18_28298_O111	Domestic pig	189,409	2018	Poland	II	[58]
MT847622.1	Pol17_31177_O81	Domestic pig	189,422	2017	Poland	II	[58]
MT847623.2	Pol19_53050_C1959/19	Domestic pig	189,413	2019	Poland	II	[58]
KJ747406.1	Kashino 04/13	Wild boar	189,387	2013/3/1	Russia	II	NA
KP843857.1	Odintsovo_02/14	Wild boar	189,333	2014/2	Russia	II	NA
MT459800.1	ASFV/Kabardino-Balkaria 19/WB-964	Wild boar	189,252	2019/3/26	Russia	II	NA
MW306190.1	ASFV/Amur 19/WB-6905	Wild boar	189,248	2019/8/29	Russia	II	[59]
MW306191.1	ASFV/Primorsky 19/WB-6723	Wild boar	189,256	2019/8/28	Russia	II	[59]
MW306192.1	ASFV/Ulyanovsk 19/WB-5699	Wild boar	189,263	2019/8/21	Russia	II	[59]
MT748042.1	ASFV/Korea/pig/PaJu1/2019	Domestic pig	190,597	2019/9/16	South Korea	II	NA
LR813622.1	Tanzania/Rukwa/2017/1	Domestic pig	183,186	2017	Tanzania	II	[60]
MW396979.1	ASFV/Timor-Leste/2019/1	Domestic pig	192,237	2019/9/27	Timor-Leste	II	[61]
MN194591.1	ASFV/Kyiv/2016/131	Domestic pig	191,911	2016/4/11	Ukraine	II	[62]
AY261365.1	Warmbaths	Tick	190,773	1987	South Africa	III	NA
AY261366.1	Warthog	Warthog	186,528	1980	Namibia	IV	NA
KM111295.1	Ken06.Bus	Domestic pig	184,368	2006	Kenya	IX	[63]
MH025916.1	R8	Domestic pig	188,627	2015	Uganda	IX	[20]
MH025917.1	R7	Domestic pig	188,628	2015	Uganda	IX	[20]
MH025918.1	R25	Domestic pig	188,630	2015	Uganda	IX	[20]
MH025919.1	N10	Domestic pig	188,611	2015	Uganda	IX	[20]
MH025920.1	R35	Domestic pig	188,629	2015	Uganda	IX	[20]
AY261364.1	Tengani 62	Domestic pig	185,689	1962	Malawi	V	NA
AY261362.1	Mkuzi 1979	Tick	192,714	1979	South Africa	VII	NA
MZ202520.1	K49	Domestic pig	189,523	1949	DR Congo	VII/I	NA
MN913970.1	Liv13/33 (OmLF2)	Tick	188,277	1983	Zambia	VII/I	[64]
AY261361.1	Malawi Lil-20/1	Tick	187,612	1983	Malawi	VIII	NA
MW856067.1	BUR/18/Rutana	Domestic pig	176,564	2018	Burundi	X	[55]
MT956648.1	Uvira B53	Domestic pig	180,916	2019/1	DR Congo	X	[65]
AY261360.1	Kenya 1950	Domestic pig	193,886	1950	Kenya	X	NA
KM111294.1	Ken05/Tk1	Tick	191,058	2005	Kenya	X	[63]
LR899131.1	Ken.rie1	NA	189,950	NA	Kenya	X	NA
AY261363.1	Pretoriuskop/96/4	Tick	190,324	1996	South Africa	XX	NA

Table S4. The length of genome, coding region sequence (CRS) and conserved central region (CCR) of the ASFV genome sequences in the curated dataset.

Acc. No	Strain	Genotype	Length	CRS.start	CRS.end	CRS.Length	A224L.start	DP238L.end	CCR.Length	Putative. ORF
AM712239.1	Benin 97/1	I	182,284	253	180,970	180,718	40,455	171,040	130,586	156
AM712240.1	OURT 88/3	I	171,719	1	171,700	171,700	31,275	161,973	130,699	157
FN557520.1	E75	I	181,187	288	181,125	180,838	39,759	170,166	130,408	163
KM102979.1	26544/OG10	I	182,906	827	182,186	181,360	42,799	173,666	130,868	164
KM262844.1	L60	I	182,362	171	182,083	181,913	40,804	171,111	130,308	163
KM262845.1	NHV	I	172,051	169	172,030	171,862	31,622	162,306	130,685	158
KP055815.1	BA71	I	180,365	1242	179,124	177,883	37,270	167,702	130,433	161
KX354450.1	47/Ss/2008	I	184,638	1193	183,447	182,255	41,792	172,032	130,241	235
MN270969.1	56/Ca/1978	I	183,636	592	183,045	182,454	41,213	171,627	130,415	235
MN270970.1	57/Ca/1979	I	183,639	592	183,048	182,457	41,215	171,629	130,415	235
MN270971.1	139/Nu/1981	I	183,645	592	183,054	182,463	41,221	171,635	130,415	235
MN270972.1	140/Or/1985	I	183,723	634	183,090	182,457	41,257	171,671	130,415	235
MN270973.1	85/Ca/1985	I	181,816	128	181,537	181,410	40,311	170,569	130,259	231
MN270974.1	141/Nu/1990	I	183,720	731	182,990	182,260	41,334	171,574	130,241	235
MN270975.1	142/Nu/1995	I	183,724	731	182,994	182,264	41,337	171,577	130,241	235
MN270976.1	60/Nu/1997	I	181,651	117	181,502	181,386	40,296	170,535	130,240	231
MN270977.1	26/Ss/2004	I	184,581	1163	183,419	182,257	41,763	172,002	130,240	235
MN270978.1	72407/Ss/2005	I	181,699	126	181,511	181,386	40,304	170,544	130,241	231
MN270979.1	97/Ot/2012	I	184,206	974	183,233	182,260	41,575	171,815	130,241	235
MN270980.1	22653/Ca/2014	I	181,869	268	181,669	181,402	40,450	170,701	130,252	231
MT932578.1	103917/18	I	181,759	1	181,672	181,672	40,354	170,594	130,241	217
MT932579.1	55234/18	I	181,761	1	181,672	181,672	40,354	170,594	130,241	217
MW723480.1	Ca1978_2	I	181,925	46	181,735	181,690	40,350	170,764	130,415	226
MW723481.1	Nu1979	I	181,859	21	181,708	181,688	40,324	170,738	130,415	224
MW723482.1	Nu1986	I	181,788	66	181,590	181,525	40,358	170,616	130,259	229
MW723483.1	Nu1990_1	I	181,751	70	181,556	181,487	40,350	170,590	130,241	229
MW723484.1	Nu1991_2	I	181,741	59	181,545	181,487	40,339	170,579	130,241	231
MW723485.1	Nu1991_3	I	181,725	41	181,527	181,487	40,321	170,561	130,241	231
MW723486.1	Nu1991_7	I	181,750	64	181,550	181,487	40,344	170,584	130,241	231
MW723487.1	Or1993_1	I	181,741	64	181,569	181,506	40,350	170,601	130,252	230
MW723488.1	Nu1993_2	I	181,748	69	181,555	181,487	40,349	170,589	130,241	231
MW723489.1	Nu1995_2	I	181,753	69	181,555	181,487	40,349	170,589	130,241	230
MW723490.1	Nu1995_3	I	181,740	59	181,552	181,494	40,347	170,586	130,240	228
MW723491.1	Nu1995_4	I	181,697	41	181,527	181,487	40,321	170,561	130,241	231

MW723492.1	4996 WB	I	181,751	69	181,553	181,485	40,349	170,587	130,239	231
MW723493.1	46830	I	181,746	67	181,553	181,487	40,347	170,587	130,241	231
MW723494.1	23221	I	181,770	69	181,567	181,499	40,349	170,601	130,253	231
MW723495.1	72398 WB	I	181,754	70	181,556	181,487	40,350	170,590	130,241	231
MW723496.1	74377	I	181,753	67	>181753	181,687	40,347	170,587	130,241	232
MW723497.1	22649	I	181,753	69	181,555	181,487	40,349	170,589	130,241	231
MW723498.1	72912 WB	I	181,804	61	181,611	181,551	40,341	170,648	130,308	231
MW723499.1	22137	I	181,751	67	181,553	181,487	40,347	170,587	130,241	231
MW723500.1	44076	I	181,755	64	181,558	181,495	40,351	170,591	130,241	231
MW736597.1	47039	I	181,738	59	181,545	181,487	40,339	170,579	130,241	231
MW736598.1	2019 WB	I	181,742	61	181,552	181,492	40,344	170,584	130,241	231
MW736599.1	98039	I	181,753	69	181,555	181,487	40,349	170,589	130,241	231
MW736600.1	30322	I	181,745	66	181,552	181,487	40,346	170,586	130,241	231
MW736601.1	49179 WB	I	181,793	64	181,605	181,542	40,344	170,639	130,296	231
MW736602.1	53706	I	181,813	69	>181813	181,745	40,349	170,644	130,296	232
MW736603.1	63525 WB	I	181,733	59	181,545	181,487	40,339	170,579	130,241	231
MW736604.1	15998	I	181,761	72	181,558	181,487	40,352	170,592	130,241	231
MW736605.1	51268	I	181,753	69	181,555	181,487	40,349	170,589	130,241	231
MW736606.1	34403	I	181,759	72	>181,759	181,688	40,352	170,592	130,241	232
MW736607.1	32516	I	181,758	69	>181,758	181,690	40,349	170,589	130,241	232
MW736608.1	113049 WB	I	181,754	70	181,556	181,487	40,350	170,590	130,241	231
MW736609.1	6396 WB	I	181,756	72	181,558	181,487	40,352	170,592	130,241	231
MW736610.1	28928	I	181,738	59	181,545	181,487	40,339	170,579	130,241	230
MW736611.1	56140	I	181,759	70	>181,759	181,690	40,350	170,590	130,241	232
MW736612.1	31208	I	181,684	25	181,514	181,490	40,305	170,546	130,242	231
MW736613.1	33747 WB	I	181,753	69	181,555	181,487	40,349	170,589	130,241	231
MW788406.1	22943_2008	I	181,733	41	181,545	181,505	40,324	170,576	130,253	228
MW800838.1	Or_1984	I	181,924	66	181,726	181,661	40,346	170,760	130,415	229
FR682468.2	Georgia 2007/1	II	190,584	416	190,169	189,754	47,287	177,417	130,131	195
KJ747406.1	Kashino 04/13	II	189,387	1156	189,203	188,048	46,331	176,451	130,121	192
KP843857.1	Odintsovo_02/14	II	189,333	853	189,183	188,331	46,305	176,404	130,100	181
LR536725.1	Belgium 2018/1	II	190,599	402	190,198	189,797	47,292	177,431	130,140	196
LR722599.1	Moldova 2017/1	II	190,598	402	190,197	189,796	47,290	177,430	130,141	195
LR722600.1	CzechRepublic 2017/1	II	190,594	402	190,179	189,778	47,287	177,427	130,141	195
LR812933.1	Arm/07/CBM/c2	II	190,145	198	189,948	189,751	46,316	176,457	130,142	192
LR813622.1	Tanzania/Rukwa/2017/1	II	183,186	178	182,721	183,009	42,541	172,604	130,064	188
LR899193.1	Germany 2020/1	II	190,592	416	190,177	189,762	47,271	177,424	130,154	190

LS478113.1	Estonia 2014	II	182,446	701	181,932	181,232	39,024	169,165	130,142	173
MG939583.1	Pol16_20186_o7	II	189,401	856	189,231	188,376	46,324	176,464	130,141	167
MG939584.1	Pol16_20538_o9	II	189,399	856	189,226	188,371	46,327	176,459	130,133	193
MG939585.1	Pol16_20540_o10	II	189,405	855	189,235	188,381	46,328	176,468	130,141	193
MG939586.1	Pol16_29413_o23	II	189,393	856	189,223	188,368	46,326	176,456	130,131	193
MG939587.1	Pol17_03029_C201	II	189,405	856	189,235	188,380	46,324	176,468	130,145	193
MG939588.1	Pol17_04461_C210	II	189,401	856	189,231	188,376	46,324	176,464	130,141	158
MG939589.1	Pol17_05838_C220	II	189,393	855	189,223	188,369	46,326	176,456	130,131	193
MH681419.1	ASFV/POL/2015/Podlaskie	II	189,394	857	189,210	188,354	46,329	176,459	130,131	193
MK128995.1	China/2018/AnhuiXCGQ	II	189,393	852	189,209	188,358	46,316	176,457	130,142	179
MK333180.1	Pig/HLJ/2018	II	189,404	856	189,234	188,379	46,327	176,467	130,141	185
MK543947.1	Belgium/Etalle/wb/2018	II	190,202	896	189,278	188,383	47,979	178,112	130,134	184
MK628478.1	ASFV/LT14/1490	II	189,399	583	189,215	188,633	47,287	177,427	130,141	189
MK645909.1	ASFV-wbBS01	II	189,394	1617	189,224	187,608	46,326	176,457	130,132	185
MK940252.1	CN/2019/InnerMongolia-AES01	II	189,403	1,620	189,219	187,600	46,326	176,467	130,142	184
MN172368.1	ASFV/pig/China/CAS19-01/2019	II	189,405	856	189,235	188,380	46,327	176,468	130,142	183
MN194591.1	ASFV/Kyiv/2016/131	II	191,911	1422	190,571	189,150	47,979	178,112	130,134	246
MN393476.1	Wuhan 2019-1	II	190,576	920	190,105	189,186	47,491	177,638	130,148	163
MN715134.1	ASFV_HU_2018	II	190,601	403	190,189	189,787	47,281	177,422	130,142	193
MT459800.1	ASFV/Kabardino-Balkaria 19/WB-964	II	189,252	720	189,068	188,349	47,287	177,417	130,131	182
MT496893.1	GZ201801	II	189,393	852	189,209	188,358	46,316	176,457	130,142	179
MT748042.1	ASFV/Korea/pig/PaJu1/2019	II	190,597	402	190,196	189,795	47,288	177,429	130,142	193
MT847620.1	Pol17_55892_C754	II	189,414	856	189,244	188,389	47,287	177,417	130,131	186
MT847621.1	Pol18_28298_O111	II	189,409	857	189,239	188,383	47,287	177,417	130,131	187
MT847622.1	Pol17_31177_O81	II	189,422	856	189,252	188,397	47,287	177,417	130,131	186
MT847623.2	Pol19_53050_C1959/19	II	189,413	856	189,244	188,389	39,423	171,081	131,659	186
MW306190.1	ASFV/Amur 19/WB-6905	II	189,248	718	189,078	188,361	46,180	176,311	130,132	178
MW306191.1	ASFV/Primorsky 19/WB-6723	II	189,256	719	189,086	188,368	46,178	176,319	130,142	180
MW306192.1	ASFV/Ulyanovsk	II	189,263	719	189,079	188,361	46,188	176,327	130,140	180

	19/WB-5699									
MW396979.1	ASFV/Timor-Leste/2019/1	II	192,237	2639	191,009	188,371	48,100	178,242	130,143	178
MW521382.1	HuB20	II	188,643	853	186,833	185,981	48,100	178,242	130,143	177
MW656282.1	Pig/Heilongjiang/HRB1/2020	II	189,355	856	189,185	188,330	46,318	176,418	130,101	185
MW856068.1	MAL/19/Karonga	II	183,325	269	182,766	182,498	42,584	172,649	130,066	182
AY261365.1	Warmbaths	III	190,773	583	190,036	189,454	46,099	176,250	130,152	186
AY261366.1	Warthog	IV	186,528	323	186,206	185,884	42,932	173,024	130,093	187
KM111295.1	Ken06.Bus	IX	184,368	446	183,696	183,251	42,799	173,666	130,868	161
MH025916.1	R8	IX	188,627	663	187,927	187,265	44,946	175,827	130,882	173
MH025917.1	R7	IX	188,628	663	187,928	187,266	44,947	175,828	130,882	174
MH025918.1	R25	IX	188,630	663	187,930	187,268	44,949	175,830	130,882	172
MH025919.1	N10	IX	188,611	663	187,911	187,249	44,935	175,813	130,879	171
MH025920.1	R35	IX	188,629	663	187,929	187,267	44,948	175,829	130,882	173
AY261364.1	Tengani 62	V	185,689	732	184,958	184,227	41,387	171,776	130,390	181
AY261362.1	Mkuzi 1979	VII	192,714	920	191,795	190,876	48,002	178,329	130,328	186
MN913970.1	Liv13/33 (OmLF2)	VII/I	188,277	1135	188,049	186,915	46,326	176,467	130,142	227
MZ202520.1	K49	VII/I	189,523	1314	188,210	186,897	46,019	176,186	130,168	189
AY261361.1	Malawi Lil-20/1	VIII	187,612	753	186,197	185,445	43,505	176,298	132,794	180
AY261360.1	Kenya 1950	X	193,886	612	193,275	192,664	49,242	180,986	131,745	184
KM111294.1	Ken05/Tk1	X	191,058	252	190,582	190,331	46,718	177,877	131,160	168
LR899131.1	Ken.rie1	X	189,950	592	189,860	189,269	47,271	177,424	130,154	174
MT956648.1	Uvira B53	X	180,916	335	180,480	180,146	39,423	171,081	131,659	158
MW856067.1	BUR/18/Rutana	X	176,564	493	175,932	175,440	35,486	164,773	129,288	144
AY261363.1	Pretoriuskop/96/4	XX/I	190,324	655	189,670	189,016	46,922	177,360	130,439	185
