# Supplementary figures and images for: Genome-Wide Diversity Analysis of African Swine Fever Virus Based on a Curated Dataset
Source: Animals (Basel). 2022 Sep 16;12(18):2446. doi: 10.3390/ani12182446 (PMC9495133; doi:10.3390/ani12182446)

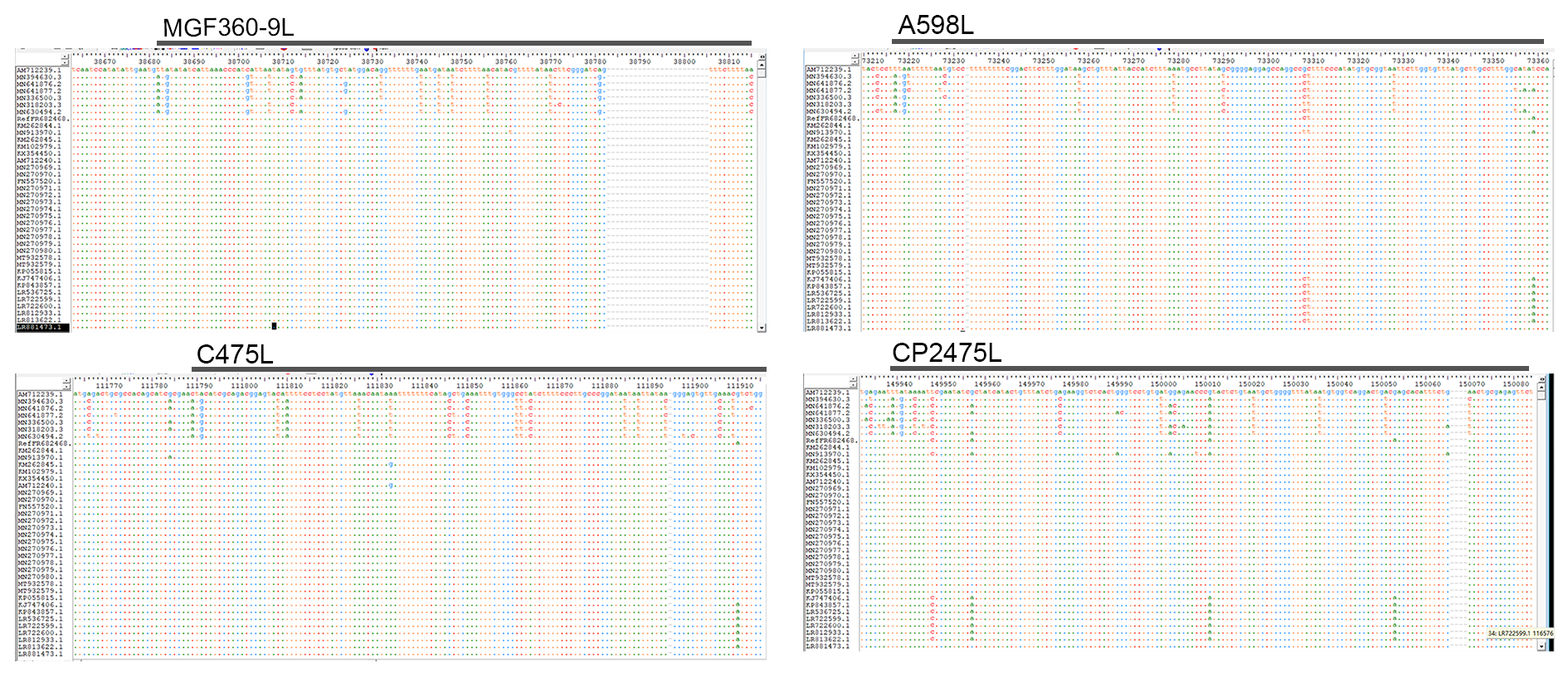

Supplement: Supplementary file 1 [file animals-12-02446-s001.zip › FigS1.MN394630Artificial.tif]
